# Impact of the COVID‐19 pandemic on the performance of endoscopy in the Tohoku region of Japan

**DOI:** 10.1002/deo2.249

**Published:** 2023-06-01

**Authors:** Katsunori Iijima, Tamotsu Matsuhashi, Yosuke Shimodaira, Tatsuya Mikami, Tetsuro Yoshimura, Shunichi Yanai, Norihiko Kudara, Tsuyotoshi Tsuji, Hiro‐o Matsushita, Hiroyuki Watanabe, Tomoyuki Koike, Katsuaki Kato, Yasuhiko Abe, Nakao Shirahata, Takuto Hikichi, Kyoko Katakura, Koji Kono, Hirotake Sakuraba, Yoshiyuki Ueno, Hiromasa Ohira, Atsushi Masamune, Takayuki Matsumoto, Shinsaku Fukuda, Japan Gastroenterological Endoscopy Society‐Tohoku

**Affiliations:** ^1^ Department of Gastroenterology Akita University Graduate School of Medicine Akita Japan; ^2^ Division of Endoscopy Hirosaki University Hospital Aomori Japan; ^3^ Department of Gastroenterology Aomori City Hospital Aomori Japan; ^4^ Department of Internal Medicine Division of Gastroenterology and Hepatology School of Medicine Iwate Medical University Iwate Japan; ^5^ Department of Internal Medicine and Gastroenterology Iwate Prefectural Ofunato Hospital Iwate Japan; ^6^ Department of Gastroenterology Akita City Hospital Akita Japan; ^7^ Digestive Disease Center, Akita Red Cross Hospital Akita Japan; ^8^ Department of Gastroenterology Ogachi Central Hospital Akita Japan; ^9^ Division of Gastroenterology Tohoku University Graduate School of Medicine Miyagi Japan; ^10^ Cancer Detection Center, Miyagi Cancer Society Miyagi Japan; ^11^ Division of Endoscopy Yamagata University Hospital Yamagata Japan; ^12^ Department of Gastroenterology Yamagata Prefectural Central Hospital Yamagata Japan; ^13^ Department of Endoscopy Fukushima Medical University Hospital Fukushima Japan; ^14^ Department of Gastroenterology Iwase general hospital Fukushima Japan; ^15^ Department of Gastrointestinal Tract Surgery Fukushima Medical University School of Medicine Fukushima Japan; ^16^ Department of Gastroenterology Hirosaki University Graduate School of Medicine Aomori Japan; ^17^ Department of Gastroenterology Faculty of Medicine Yamagata University Yamagata Japan; ^18^ Department of Gastroenterology Fukushima Medical University School of Medicine Fukushima Japan

**Keywords:** colonoscopy, colorectal cancers, COVID‐19, endoscopy, gastric cancers

## Abstract

**Objectives:**

The whole picture of the disturbance in endoscopy performance caused by the coronavirus disease 2019 (COVID‐19) pandemic in Japan remains to be clarified. Therefore, the Japan Gastroenterological Endoscopy Society‐Tohoku conducted this questionnaire survey in Tohoku region of Japan.

**Methods:**

A questionnaire on the number of diagnostic endoscopy procedures and resulting diagnosed cancers in 2019 and 2020 was sent to all guidance/guidance cooperation hospitals in the Japan Gastroenterological Endoscopy Society who worked in the Tohoku region. The percentage change was calculated by comparing the numbers in 2020 with those in 2019 (the pre‐COVID‐19 period).

**Results:**

Among the applicable 89 guidance/guidance cooperation hospitals, 83 (94%) returned the questionnaire. The number of endoscopy procedures promptly decreased to the nadir in April and May 2020 (during the first state of emergency in Japan); however, it recovered relatively quickly, within a few months after the state of emergency was lifted. Consequently, the annual reduction in the number of endoscopy procedures in 2020 (in comparison to 2019) was 10.1% for esophagogastroduodenoscopy and 7.9% for colonoscopy. The reduction in the number of diagnostic endoscopy procedures led to a 5.5% reduction in esophagogastric cancer and 2.7% in colorectal cancer.

**Conclusions:**

This is the most comprehensive survey on the impact of the COVID‐19 pandemic on the performance of endoscopy and the resulting diagnosis of cancer in Japan. Understanding the magnitude of the decline in endoscopic examinations and cancer detection due to the pandemic is critical to understanding how many people will ultimately be affected and establishing a strategy for providing endoscopy during national emergencies.

## INTRODUCTION

Coronavirus disease 2019 (COVID‐19) was first identified in Wuhan, China in December 2019, and has subsequently spread worldwide in 2020, leading to an ongoing global pandemic. The pandemic placed a serious burden on the overall healthcare system, not only the resources used directly in the treatment of COVID‐19 but also the resources used for other serious life‐threatening diseases, including cancer.^1^, ^2^, ^3^, ^4^ With increasing awareness that gastrointestinal (GI) endoscopy is an aerosol‐generating procedure and concerns about fecal shedding of severe acute respiratory syndrome coronavirus 2,^5^, ^6^ the procedures were suspended worldwide, leading to a substantial decrease in the diagnosis of GI cancers.^1^, ^2^, ^3^, ^4^ These disruptions in the diagnostic procedure for detecting GI cancers due to the pandemic are expected to cause delays in the diagnosis of cancer and ultimately result in an excess of advanced cancer diagnoses and deaths in the coming years.^1^

In Japan, the first state of emergency to prevent the spread of COVID‐19 infection was declared on April 7, 2020, and lasted until May 25, 2020. At the same time, the Japan Gastroenterological Endoscopy Society (JGES) warned about the potential transmission of COVID‐19 infection at endoscopic examinations and provided recommendations to assure the highest level of protection against COVID‐19 for both patients and healthcare personnel.^6^ Since then, many hospitals refrained from conducting endoscopic examinations throughout the country, irrespective of the varying regional infection rate. The JGES shortly announced the nationwide short‐term (a few months) change in the number of endoscopy procedures after the recommendation in October 2020;^7^ however, the effect of the disturbance on the long‐term annual outcome remains to be clarified. Although some studies reported changes in numbers at a single hospital or selected hospitals during the pandemic,^8^, ^9^, ^10^ a survey targeting unselected medical institutes in a given area is important to understand the whole picture of the disturbance in the endoscopy caused by the pandemic.

In this study, to grasp the actual situation of digestive endoscopy performance during the pandemic in a given area (Tohoku region in Japan), JGES‐Tohoku conducted the present questionnaire survey on the number of diagnostic endoscopy procedures and corresponding cancer diagnoses.

## METHODS

This questionnaire survey was initiated and organized by JGES‐Tohoku. In August 2022, a structured questionnaire was sent to the chief physicians of all guidance/guidance cooperation hospitals (G/GC‐Hs) of the JGSE in the Tohoku region, Japan, or to all branch councilor physicians for the JGSE who work at facilities other than G/GC‐Hs (mainly [76%] private clinics in the region). Then, the questionnaires were returned to the JGSE‐Tohoku headquarters at Akita University for their analysis. If there was no response to the initial invitation, a further reminder was sent by email in October 2022. The study protocol was reviewed and approved collectively by the ethics committee of Akita University (2858).

### Questionnaire

The questionnaires consisted of 2 parts: one was on the number of diagnostic endoscopy procedures performed from January 2019 to March 2021, and the other was on the number of resulting cancer diagnoses in 2019 and 2020 (Figure S1). All participating institutes were asked to report information on the monthly numbers of diagnostic esophagogastroduodenoscopy (EGD) and colonoscopy (CS), excluding therapeutic endoscopy. Furthermore, G/GC‐Hs of the JGSE were asked to report information on the yearly number and stage of diagnosed esophageal, gastric, and colorectal cancers. For the diagnosis of cancer, endoscopically suspected lesions required histological confirmation. Adenomas were excluded from the analysis. The cancer stages were roughly classified into superficial (confined to the mucosal or submucosal layers) and advanced (invasion to at least the muscularis propria) mainly based on endoscopic evaluations by endoscopists by the JGES.^11^, ^12^

### Statistical analyses

The percentage changes in the yearly and monthly figures were calculated by comparing the numbers in 2020 with the comparable numbers in 2019 (the pre‐COVID‐19 baseline period). Esophageal and gastric cancers were combined as esophagogastric cancer (EGC), as both cancers are detected by EGD. In the analysis of the number of cancers, counts from health check‐up institutes were excluded from the sum to avoid the duplication of cases that were referred to other hospitals.

## RESULTS

**FIGURE 1:**
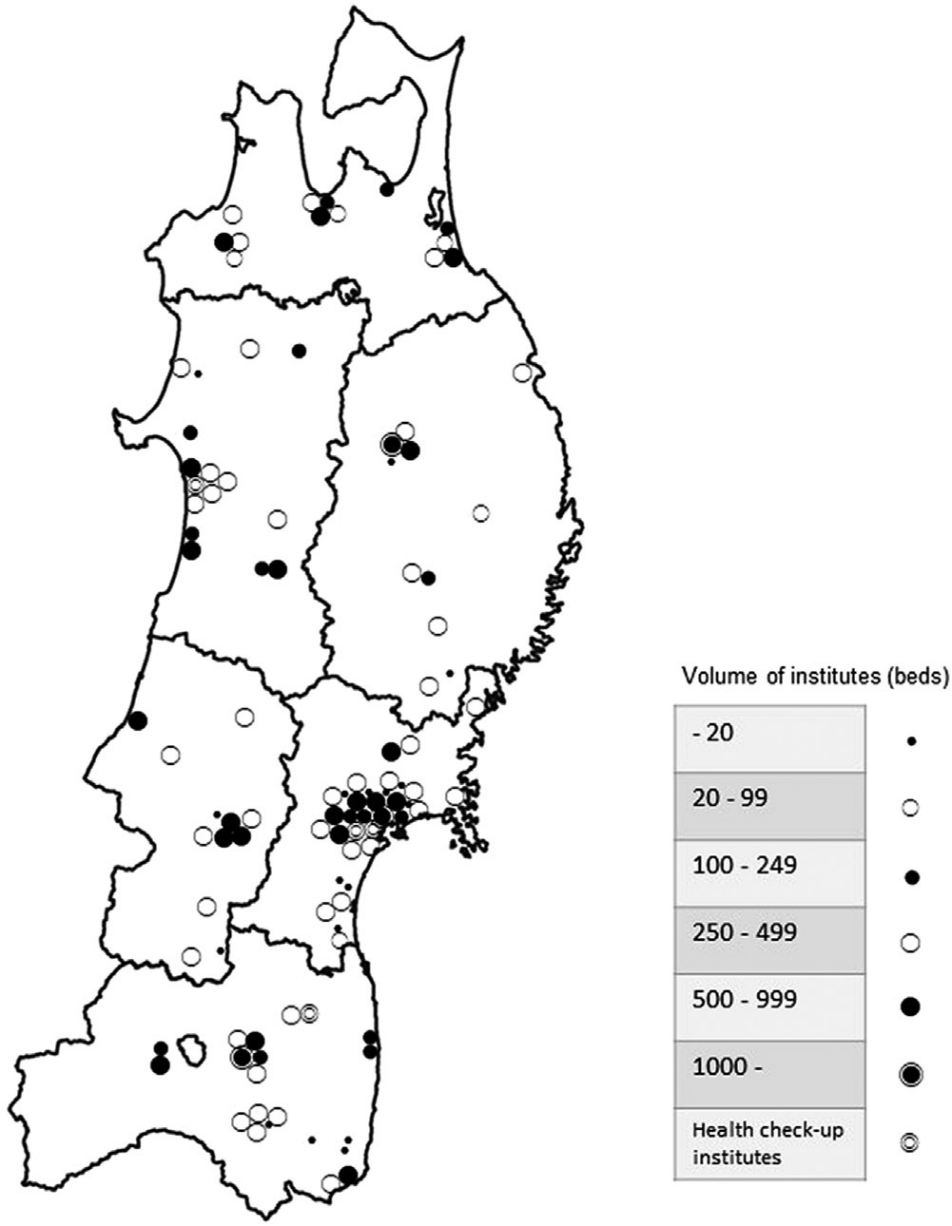
Distribution of participating institutes in the Tohoku region, Japan.

The questionnaire was sent to the 89 applicable G/GC‐Hs and 85 branch councilors who work mainly at clinics. Among the G/GC‐Hs, 3 declined to participate in the study, and three did not show any response, and then, 83 G/GC‐Hs (94%) returned the questionnaire consisting of at least the first part (number of endoscopy procedures). Further, 63 hospitals and one health check‐up institute (72%) completed both parts of the questionnaire (number of endoscopy procedures and cancer diagnoses). Twenty of the branch councilors (33%) responded to the questionnaire. Consequently, the participating institutes included hospitals of various sizes distributed throughout the 6 prefectures of the Tohoku region (Table 1 and Figure 1).

**TABLE 1:** Participating institutes of this study.

	*N* (participation/non‐participation)	
Type of institutes	The first part of questionnaire (No. of endoscopy)	The second part of questionnaire (No. of cancer)
Guidance/guidance cooperation hospitals		
Volume of hospital (bed number)		
20–99	1/1	0/2
100–249	13/0	8/5
250–499	46/4	38/12
500–999	19/1	14/6
1000	3/0	3/0
Health check‐up institutes	1/0	1/0
Total	83/6	64/25
Other branch councilors	28/57	N/A

**FIGURE 2:**
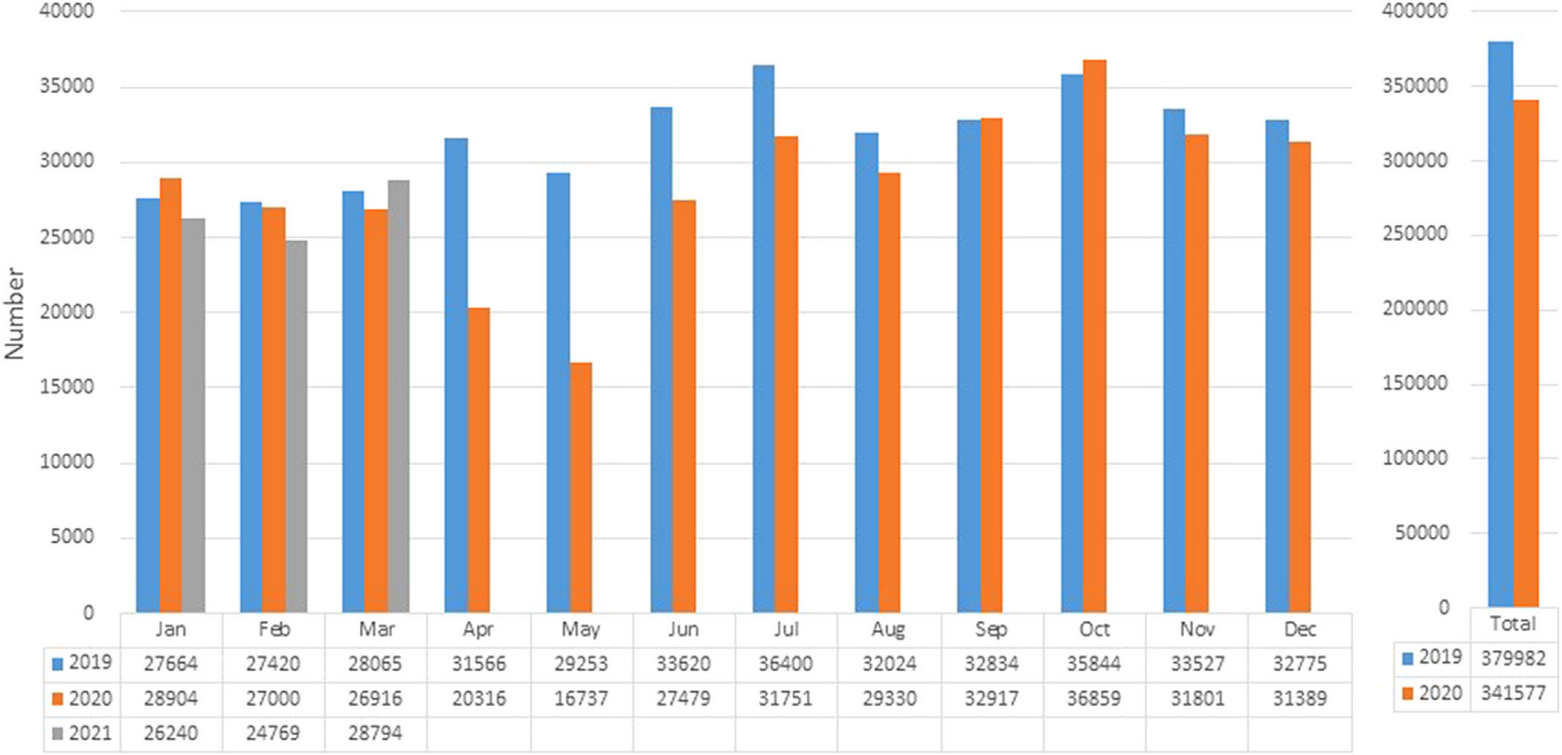
The change in the number of esophagogastroduodenoscopy procedures performed in all participating institutes before and during the coronavirus disease 2019 (COVID‐19) pandemic. The monthly and total yearly numbers of esophagogastroduodenoscopy procedures are represented in bar graphs.

**FIGURE 3:**
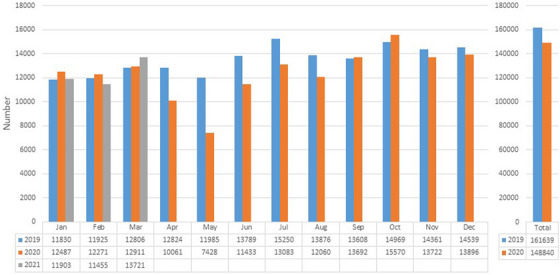
The change in the number of colonoscopy procedures performed in all participating institutes before and during the coronavirus disease 2019 (COVID‐19) pandemic. The monthly and total yearly numbers of colonoscopy procedures are represented in bar graphs.

The changes in the number of EGD and CS procedures performed in all participating institutes during the study period are shown in Figures 2 and 3, respectively. Coinciding with the commencement of the first state of emergency, the number of EGD procedures promptly dropped by 35.6% in April 2020 in comparison to the pre‐COVID period, and reached the nadir in the next month with a 42.8% decrease from 29,253 in 2019 to 16,737 in 2020. Thereafter, soon after the termination of the first state of emergency, the number of EGD procedures quickly increased in June 2020, making a nearly full recovery to the pre‐pandemic level by July–August (Figure 2). Eventually, the total number of diagnostic EGD procedures performed in 2020 was 341,577, and was 10.1% lower than that in 2019 (*n* = 379,982). Then, although there was another wave of infection, the number of EGD procedures was unchanged, even in the second state of emergency (January–March 2021), when the number of infections sharply increased in comparison to previous waves of infection in Tohoku region.

The number of CS procedures showed a similar trend to that observed in EGD procedures, although the overall change was relatively mild in comparison to EGD procedures. The decrease in the number of CS procedures started in April 2020 and reached a nadir of 38.0% decrease in the following month. Then, after the number recovered, it did not decrease again during the study period (Figure 3). Consequently, the total number of CS procedures showed a 7.9% decrease in 2020 in comparison to 2019 (148,840 vs. 161,639). The overall trends in the number of EGD or CS procedures were largely similar, even when only 63 hospitals that reported the number of cancer cases were included (Figures S2 and S3).

When dividing the participating hospitals into 3 groups according to the number of beds, the magnitude of decrease in the number of EGD procedures seemed to be smaller in low‐volume hospitals (20–249 beds) in comparison to higher‐volume hospitals (250–499 beds or ≥500 beds); the decrease in 2020 relative to 2019 was −6.6% (20–249 beds), −11.3% (250–499 beds), and −11.6% (≥500 beds). Meanwhile, there was no obvious trend in the magnitude of the decrease in the number of CS procedures according to the volume of the hospitals (9.4% [20–249 beds], −7.3% [250–499 beds], and −8.8% [≥500 beds]). In addition, the magnitude of the decrease in the number of endoscopies also varied depending on the volume of endoscopy in each institute (Table 2).

**TABLE 2:** Magnitude of decrease in the yearly number of esophagogastroduodenoscopies and colonoscopies due to the coronavirus disease 2019 (COVID‐19) pandemic by hospital size.

	Number of participating institutes	EGDs	CSs
Number of beds			
20–249	14	−6.6%	−9.4%
250–499	46	−11.3%	−7.3%
500–	22	−11.6%	−8.8%
Number of total endoscopies (EGDs + CSs) in 2019			
–2999	47	−5.1%	−3.3%
3000–6999	43	−13.8%	−8.0%
7000	21	−7.4%	−7.7%

Abbreviations: CS, colonoscopy; EGD, esophagogastroduodenoscopy.

**FIGURE 4:**
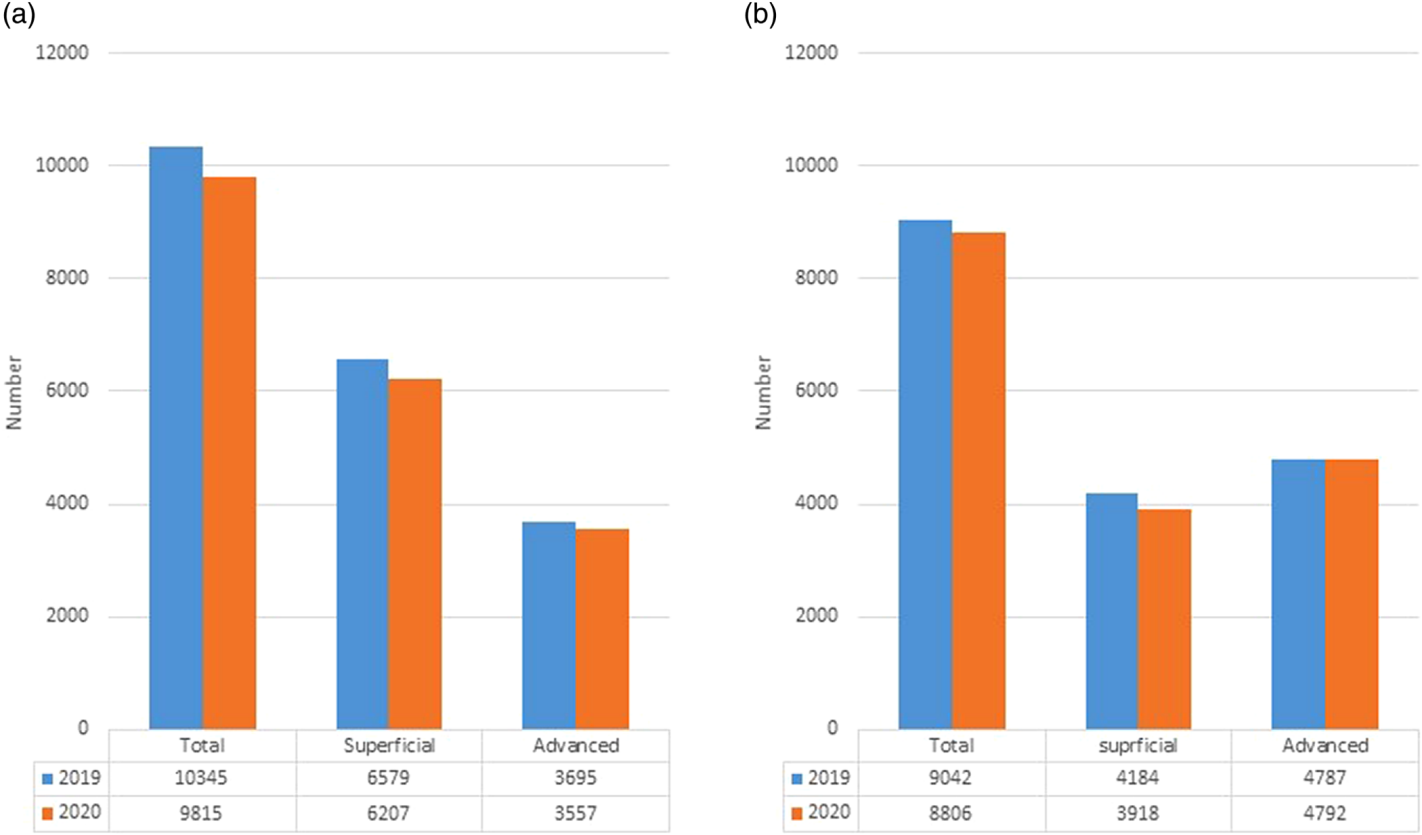
The annual change in the number of total and superficial/advanced‐stage esophagogastric cancers (a) and colorectal cancers (b) diagnosed in 2019 and 2020.

In 2020, a total of 9815 EGCs were diagnosed based on EGD procedures in 63 hospitals, a 5.1% reduction in comparison to the previous year (n = 10,345). The reduction of EGCs diagnoses was more prominent in superficial type cases in comparison to advanced type cases (−5.7% vs. −3.7%). Meanwhile, the magnitude of the reduction in the number of colorectal cancer (CRC) cases diagnosed by CS in the 63 hospitals were somewhat smaller in comparison to EGCs; a total of 8806 CRCs were diagnosed, a reduction of 2.7% in comparison to the previous year (n = 9042). Interestingly, the reduction of CRC diagnoses was only observed in superficial type CRC (6.4% reduction), while the number of advanced‐type CRC diagnoses marginally increased in 2020 in comparison to 2019. (Figure 4). Additional questionnaires regarding monthly number of diagnosed cancers were sent to the participating hospital, and the outcomes from 13 institutes were shown in Figure S4.

**FIGURE 5:**
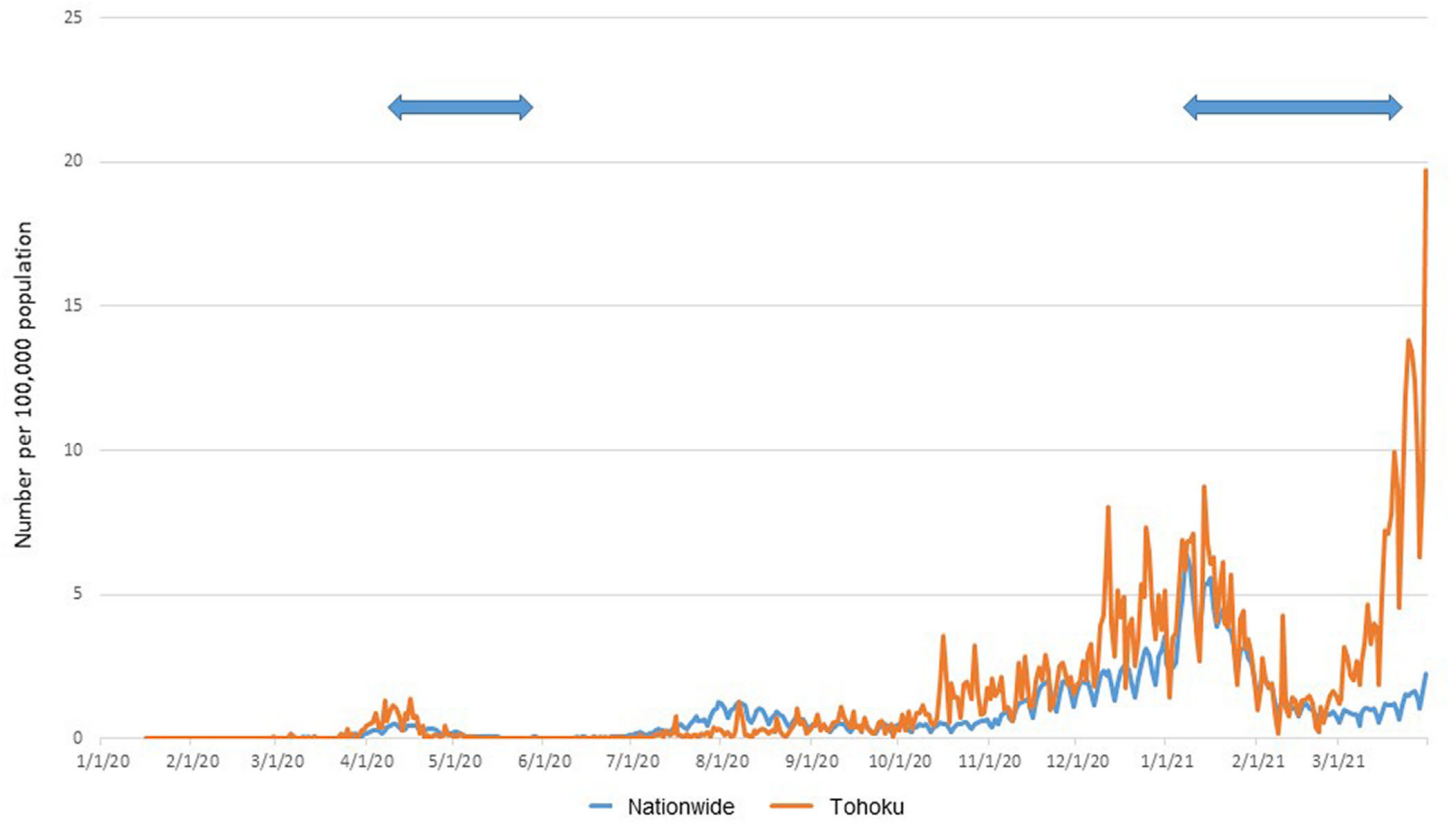
Plot diagrams of the number of coronavirus disease 2019 (COVID‐19) cases per 100,000 population throughout the whole country and the Tohoku region. The data was based on the open data of the Ministry of Health, Labour and Welfare, Japan. Arrows indicate periods in which a state of emergency was declared for COVID‐19 in Japan.

As a reference, plot diagrams on the number of COVID‐19 cases per 100,000 population throughout the whole country and Tohoku region^13^ are shown in Figure 5.

## DISCUSSION

This JGES‐Tohoku‐led survey successfully revealed the big picture of the impact of the COVID‐19 pandemic on the endoscopy performance in a given area of Japan (the Tohoku region) by collecting data from more than 90% G/GC‐Hs of the JGSE in the Tohoku region. In addition, we could directly show the relationship between the decrease in the number of endoscopy procedures during the pandemic and the reduction in the number of GI cancers diagnosed by endoscopy in the area.

Regarding the reduction in the number of endoscopy procedures during the pandemic in Japan, the JGES announced the nationwide short‐term (a few months) trends;^7^ however, the annual decrease, which is responsible for the annual reduction in the number of diagnosed GI cancers, remains to be clarified. More recently, the annual reduction in the number of endoscopy procedures during the pandemic was reported using the Japan endoscopy database, a nationwide endoscopic data repository.^14^ However, the outcome was obtained from 60 highly selected high‐volume institutes throughout Japan,^14^ and whether this actually reflected the situation of endoscopy performance during the pandemic in Japan remains unknown. Such consideration is important, since the influence of the pandemic on endoscopy performance may differ depending on the volumes of the institutes, as shown in the current study.

In the current study, in corporation with almost all G/GC‐Hs of the JGSE‐Tohoku, which included various hospitals that performed endoscopy procedures, we clarified an annual reduction of the number of endoscopy procedures during the pandemic. The number of endoscopy procedures promptly decreased to the nadir in April and May 2020 (during the first state of emergency in Japan); however, it quickly recovered after the state of emergency was lifted. Since then, it has not decreased, even though a series of more intense surges of infection subsequently occurred throughout the country, including the Tohoku region (e.g., January– March 2021). Consequently, in comparison to the pre‐pandemic value is 2019, the annual reduction in the number of endoscopy procedures in 2020 was 10.1% for EGD procedures and 7.9% for CS procedures. Although the prevalence of COVID‐19 infection could be quite different depending on the location within the country, especially in the initial state of emergency, the short‐term survey by the JGES indicated that the endoscopic procedures were similarly interrupted all over the country.^7^ Hence, the current outcomes from the Tohoku region may be representative of the bigger picture of the nationwide outcomes during the pandemic.

There have been some reports on the reduction of diagnostic endoscopy during the COVID‐19 pandemic—mainly from Western countries—in which the reduction in the number of procedures was as great as 80%–95%, and where the disruption lasted the entire year of 2020.^2^, ^3^, ^4^, ^15^ In addition, a recent report on the global impact of the COVID‐19 pandemic on endoscopy in 35 countries demonstrated, on average, a 55% annual reduction in EGD procedures and a 45% reduction in CS procedures in 2020.^16^ Thus, the impact of the pandemic on the performance of endoscopy seemed to be much milder in Japan in comparison to other countries. There are several possible explanations for the difference. First, the number of infected subjects and deaths was much lower in Japan in comparison to other countries, especially in the initial phase of the pandemic. Second, the development and widespread use of various infection protection devices (e.g., mouthpieces, face masks, and face shields) may have had some effect on the maintenance of the number of endoscopy procedures in Japan, especially for EGD procedures.^17^, ^18^, ^19^ Indeed, all the responding institutes followed the recommendation for the personal protective equipment proposed by JGES.^6^

Since all GI cancers are exclusively diagnosed by endoscopic examinations with targeted biopsies, the potential decrease in the number of endoscopy procedures performed during the pandemic should inevitably lead to a decrease in the number of diagnosed GI cancers; this scenario is already supported by solid data from other countries.^2^, ^3^, ^4^ Indeed, we found that the reduction in the number of diagnostic endoscopy procedures led to a decrease in the number of diagnosed GI cancers in this study. In addition, the magnitude of reduction for superficial cancers was more prominent for both EGCs and CRCs than for advanced cases, probably due to the reduction of screening endoscopy for asymptomatic individuals.

Interestingly, the magnitude of the annual reduction in the total number of EGCs from 2019 to 2020 was twice that observed in CRCs (5.5% vs. 2.7%); this phenomenon is consistent with a nationwide survey from National Cancer Japan.^20^ The temporal declining trend in EGCs in comparison to CRCs in Japan may partly explain the prominent annual reduction in EGC diagnoses. However, a simulation model predicted a 1.0% annual reduction rate for the sum of EGC (esophageal cancers plus gastric cancers) in Japan from 2015–2019;^21^ hence, the temporal trend alone could not fully explain the phenomenon observed in this study. Alternatively, the more marked reduction of EGD procedures in comparison to CS procedures in the current study (10.1% vs. 7.9%) may be at least partly responsible for the prominent reduction of EGC diagnoses in comparison to CRC diagnoses. The more marked reduction of EGD procedures relative to CS procedures seemed to be consistently reported from other studies in Japan and other countries,^8^, ^9^, ^16^, ^22^, ^23^ probably due to concerns about the potential transmission of infection through coughing and the subsequent emission of droplets during EGD.^24^, ^25^ In Western countries, where CRCs are much more prevalent than EGCs, most researchers have mainly focused on the impact of COVID‐19 on the number of CS procedures and the resulting number of CRC diagnoses.^3^, ^4^, ^15^, ^26^ On the other hand, in Asia—including Japan—EGCs still show a similar prevalence to CRCs, as shown in the current study. Hence, maintaining the performance of EGD and CS procedures, despite the ongoing pandemic, is important for the management of total GI cancers in Japan.

The current finding that the number of advanced‐stage CRCs increased during the pandemic while the total number of CRCs decreased is consistent with some previous studies.^10^, ^27^ A recent study using a simulation model in Hong Kong demonstrated that 4.6% of patients with gastric cancer and 6.4% of patients with CRCs will shift to a higher stage with a 6‐month delay due to the COVID‐19 pandemic.^2^ Thus, the influence of the delayed diagnosis of cancers may have already appeared within the first year of the pandemic 2020. We need to watch the subsequent trend carefully in the coming years when the impact of delayed diagnoses is likely to become more apparent.^28^

This is the first study to comprehensively investigate the impact of COVID‐19 on endoscopy performance and the resulting diagnosis of GI cancers in a defined area of Japan, which enables us to make international comparisons on the topic. However, the present study was associated with some limitations. First, we simplified the questionnaire items as much as possible to achieve a high response rate, however, in exchange for it, information on each patient is lacking. Hence, we could not analyze factors associated with a decrease in the number of endoscopies (e.g., age, sex, comorbidities, purposes for examination, and reasons for the cancellation). Second, the participation rate of branch councilors who mainly work at clinics was low in this study, although their contribution to the overall picture would be expected to be small. Third, not all participating G/GC‐Hs reported the number of diagnosed cancers, although the trends in the number of endoscopy procedures were largely similar between hospitals that did and did not report the number of diagnosed cancers. Finally, the number of cancers may have included duplicated cases due to referrals between participating hospitals. However, clinics and health check‐up institutes were not included in the analysis on the number of cancers, and most of the remaining G/GC‐Hs offer cancer treatment without a referral; thus, the number of duplicated cases is expected to be low.

In conclusion, we found a substantial reduction in the number of diagnostic endoscopy procedures and the corresponding diagnosed GI cancers during the COVID‐19 pandemic using a survey that covered the majority of hospitals that performed endoscopy procedures in the Tohoku region, Japan. Since virtually all GI cancers are diagnosed by endoscopy, understanding the magnitude of the decline in endoscopic examinations and cancer detection due to the pandemic is critical to understanding how many people will ultimately be affected and establishing a strategy for providing endoscopy during national emergencies.

## CONFLICT OF INTEREST STATEMENT

None

REFERENCES1

Dinmohamed
AG
, 
Visser
O
, 
Verhoeven
RHA

*et al*. Fewer cancer diagnoses during the COVID‐19 epidemic in the Netherlands. Lancet Oncol
2020; 21: 750–1.3235940310.1016/S1470-2045(20)30265-5PMC72521802

Lui
TKL
, 
Leung
K
, 
Guo
CG
, 
Tsui
VWM
, 
Wu
JT
, 
Leung
WK
. Impacts of the coronavirus 2019 pandemic on gastrointestinal endoscopy volume and diagnosis of gastric and colorectal cancers: A population‐based study. Gastroenterology
2020; 159: 1164–66.e3.3242522810.1053/j.gastro.2020.05.037PMC72301393

Khan
A
, 
Bilal
M
, 
Morrow
V
, 
Cooper
G
, 
Thakkar
S
, 
Singh
S
. Impact of the coronavirus disease 2019 pandemic on gastrointestinal procedures and cancers in the United States: A multicenter research network study. Gastroenterology
2021; 160: 2602–4.3366238810.1053/j.gastro.2021.02.055PMC79195134

Rutter
MD
, 
Brookes
M
, 
Lee
TJ
, 
Rogers
P
, 
Sharp
L
. Impact of the COVID‐19 pandemic on UK endoscopic activity and cancer detection: A national endoscopy database analysis. Gut
2021; 70: 537–43.3269060210.1136/gutjnl-2020-3221795
Endoscopy activity and COVID‐19: BSG and JAG guidance. *British Society of Gastroenterology*. Available from: https://www.bsg.org.uk/covid‐19‐advice/endoscopy‐activity‐and‐covid‐19‐bsg‐and‐jag‐guidance/
6

Irisawa
A
, 
Furuta
T
, 
Matsumoto
T

*et al.*
Gastrointestinal endoscopy in the era of the acute pandemic of coronavirus disease 2019: Recommendations by Japan Gastroenterological Endoscopy Society (Issued on April 9th, 2020). Dig Endosc
2020; 32: 648–50.3233594610.1111/den.13703PMC72671597
Japan Gastroenterological Endoscopy Society
. Available from: https://www.jges.net/medical/covid‐19‐qs. Accessed October 7, 2020.8

Saito
H
, 
Igarashi
K
, 
Murakami
F

*et al.*
Impact of COVID‐19 on the endoscopy department since the early phase of the pandemic in 2020: A questionnaire study among patients with canceled examinations at a single Japanese institution. Asian J Endosc Surg
2023; 16: 58–67.3605889810.1111/ases.13123PMC95386909

Maruyama
H
, 
Hosomi
S
, 
Nebiki
H

*et al*. Gastrointestinal endoscopic practice during COVID‐19 pandemic: A multi‐institutional survey. Rom J Intern Med
2021; 59: 166–73.3382681210.2478/rjim-2020-003810

Iijima
K
, 
Jin
M
, 
Miura
M

*et al*. Disturbance of gastrointestinal cancers diagnoses by the COVID‐19 pandemic in a depopulated area of Japan: A population‐based study in Akita Prefecture. Tohoku J Exp Med
2022; 257: 65–71.3538790710.1620/tjem.2022.J02011

Ono
H
, 
Yao
K
, 
Fujishiro
M

*et al*. Guidelines for endoscopic submucosal dissection and endoscopic mucosal resection for early gastric cancer. Dig Endosc
2021; 33: 4–20.3310711510.1111/den.1388312

Hashiguchi
Y
, 
Muro
K
, 
Saito
Y

*et al*. Japanese Society for Cancer of the Colon and Rectum (JSCCR) guidelines 2019 for the treatment of colorectal cancer. Int J Clin Oncol
2020; 25: 1–42.3120352710.1007/s10147-019-01485-zPMC694673813
Ministry of Health, Labour and Welfare, Japan
. 2023. Available from: https://www.mhlw.go.jp/stf/covid‐19/open‐data.html Accessed March 1^st^, 2022.14
Japan Gastroenterological Endoscopy Society.
Available from: https://www.jges.net/medical/content/jed_whitepaper Accessed March 1^st^, 2022.15

Lee
JK
, 
Lam
AY
, 
Jensen
CD

*et al.*
Impact of the COVID‐19 pandemic on fecal immunochemical testing, colonoscopy services, and colorectal neoplasia detection in a large United States community‐based population. Gastroenterology
2022; 163: 723–31.3558065510.1053/j.gastro.2022.05.014PMC910710116

Srinivasan
S
, 
Sundaram
S
, 
Emura
F

*et al*. Ongoing global impact of the COVID‐19 pandemic on endoscopy: A subsequent international survey of 121 centers from 35 countries. Gastroenterology
2022; 162: 328–30.e.3456347410.1053/j.gastro.2021.09.042PMC845946417

Onoyama
T
, 
Fujii
M
, 
Isomoto
H
. Useful face‐protective shield “ORIGAMI” for gastrointestinal endoscopy during the COVID‐19 pandemic. Dig Endosc
2020; 32: 998.3257891010.1111/den.13780PMC736213718

Endo
H
, 
Koike
T
, 
Masamune
A
. Novel device for preventing diffusion of aerosol droplets from subjects undergoing esophagogastroduodenoscopy during COVID‐19 pandemic. Dig Endosc
2020; 32: e140–1.3269699610.1111/den.13772PMC740510819

Hikichi
T
, 
Nakamura
J
, 
Hamada
K
, 
Nemoto
D
. A novel endoscopic mouthpiece for COVID‐19 prevention. Clin Endosc
2022; 55: 160–2.3464941910.5946/ce.2021.172PMC883142120

Okuyama
A
, 
Watabe
M
, 
Makoshi
R
, 
Takahashi
H
, 
Tsukada
Y
, 
Higashi
T
. Impact of the COVID‐19 pandemic on the diagnosis of cancer in Japan: Analysis of hospital‐based cancer registries. Jpn J Clin Oncol
2022; 52: 1215–24.3590932510.1093/jjco/hyac129PMC938460021
National Cancer Center Japan.
2021 Available from: https://ganjoho.jp/public/index.html Accessed November 26, 2021.22

Lantinga
MA
, 
Theunissen
F
, 
Ter Borg
PCJ

*et al*. Impact of the COVID‐19 pandemic on gastrointestinal endoscopy in the Netherlands: Analysis of a prospective endoscopy database. Endoscopy
2021; 53: 166–70.3308063010.1055/a-1272-3788PMC786903523

Chai
N
, 
Tang
X
, 
Linghu
E

*et al*. The influence of the COVID‐19 epidemic on the gastrointestinal endoscopy practice in China: A national survey. Surg Endosc
2021; 35: 6524–31.3317918110.1007/s00464-020-08149-4PMC765737824

Sagami
R
, 
Nishikiori
H
, 
Sato
T

*et al*. Aerosols produced by upper gastrointestinal endoscopy: A quantitative evaluation. Am J Gastroenterol
2021; 116: 202–5.3307974710.14309/ajg.000000000000098325

Gregson
FKA
, 
Shrimpton
AJ
, 
Hamilton
F

*et al.*
Identification of the source events for aerosol generation during oesophago‐gastro‐duodenoscopy. Gut
2022; 71: 871–8.3418784410.1136/gutjnl-2021-32458826

Morris
EJA
, 
Goldacre
R
, 
Spata
E

*et al*. Impact of the COVID‐19 pandemic on the detection and management of colorectal cancer in England: A population‐based study. Lancet Gastroenterol Hepatol
2021; 6: 199–208.3345376310.1016/S2468-1253(21)00005-4PMC780890127

Kuzuu
K
, 
Misawa
N
, 
Ashikari
K

*et al*. Gastrointestinal cancer stage at diagnosis before and during the COVID‐19 pandemic in Japan. JAMA Netw Open
2021; 4: e2126334.3454636810.1001/jamanetworkopen.2021.26334PMC845638628

Iijima
K
, 
Shimodaira
Y
, 
Watanabe
K

*et al.*
A follow‐up report on the diagnosis of gastrointestinal cancer during the COVID‐19 pandemic in Akita prefecture, Japan in 2021. Tohoku J Exp Med
2023; 259: 301–6.3669698110.1620/tjem.2023.J007

## Supporting information

Figure S1 Questionnaire items used in this study.

Figure S2 The change in the number of esophagogastroduodenoscopy procedures performed at 63 participating hospitals that completed the questionnaire.

Figure S3 The change in the number of colonoscopy procedures performed at 63 participating hospitals that completed the questionnaire.

Figure S4 Monthly number of diagnosed esophagogastric cancers (a) and colorectal cancers (b) in 13 institutes.
