# Supplementary figures and images for: Impact of the COVID‐19 pandemic on the performance of endoscopy in the Tohoku region of Japan
Source: DEN Open. 2023 Jun 1;4(1):e249. doi: 10.1002/deo2.249 (PMC10235797; doi:10.1002/deo2.249)

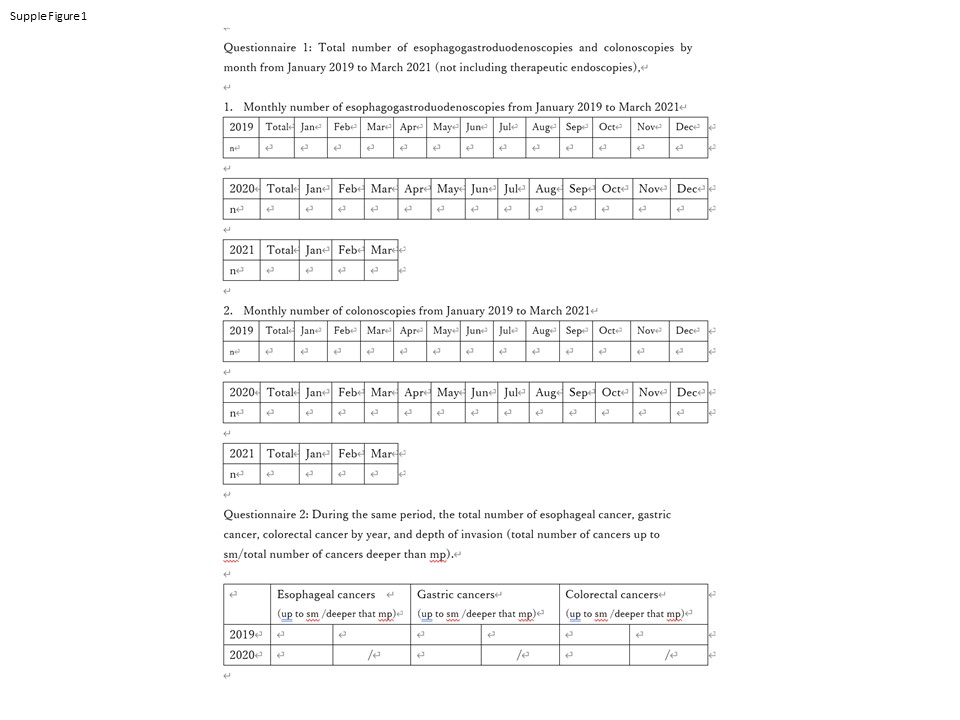

Supplement: Supplementary file 1 — Figure S1 Questionnaire items used in this study. [file DEO2-4-e249-s002.JPG]

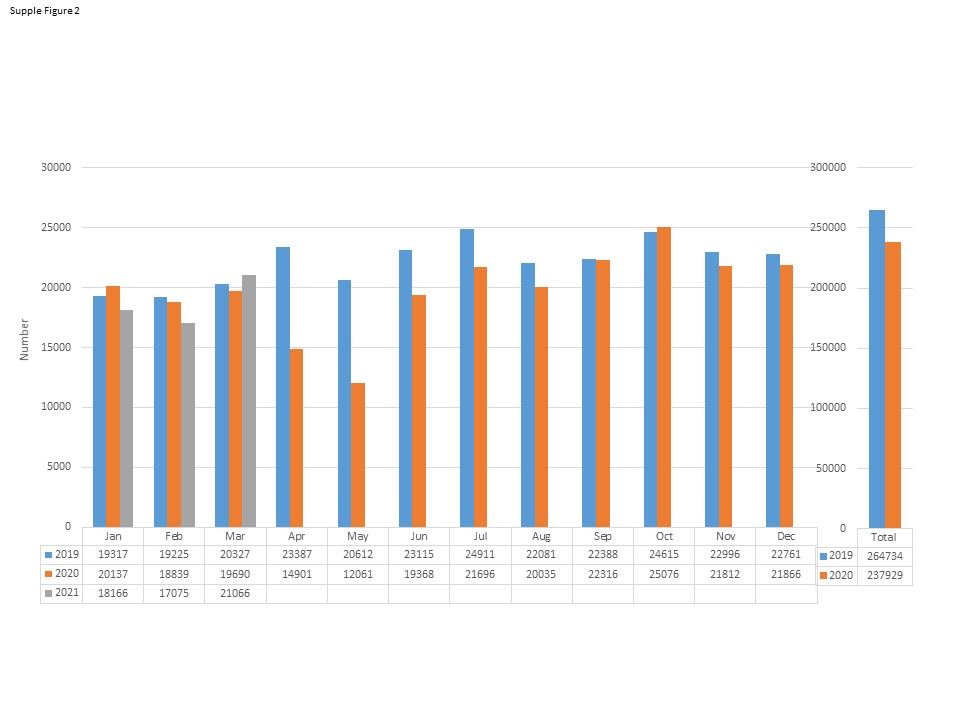

Supplement: Supplementary file 2 — Figure S2 The change in the number of esophagogastroduodenoscopy procedures performed at 63 participating hospitals that completed the questionnaire. [file DEO2-4-e249-s004.JPG]

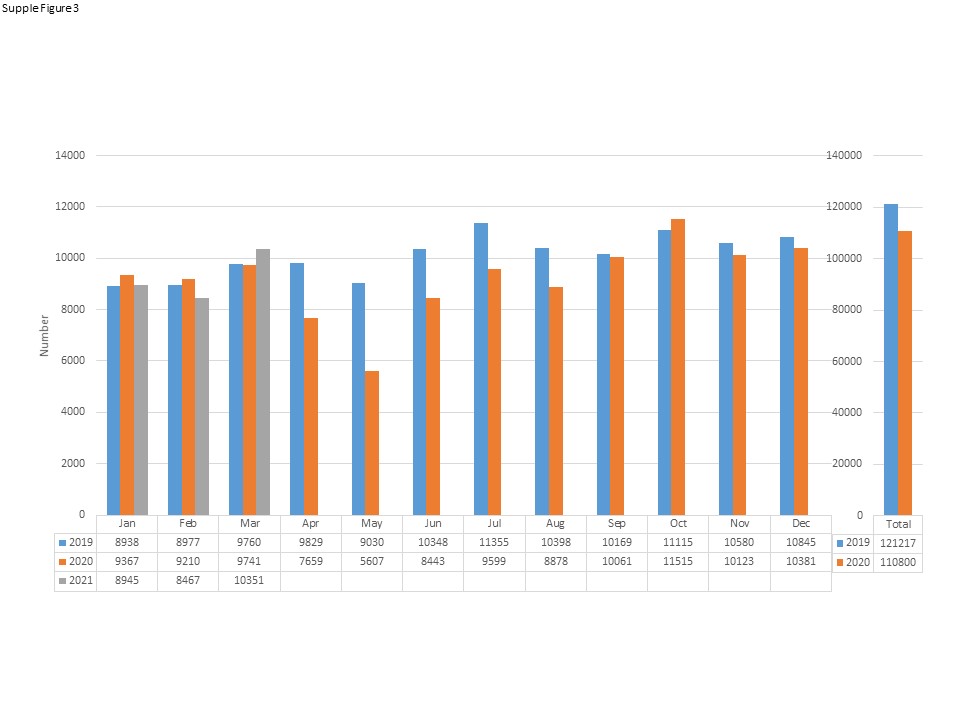

Supplement: Supplementary file 3 — Figure S3 The change in the number of colonoscopy procedures performed at 63 participating hospitals that completed the questionnaire. [file DEO2-4-e249-s003.JPG]

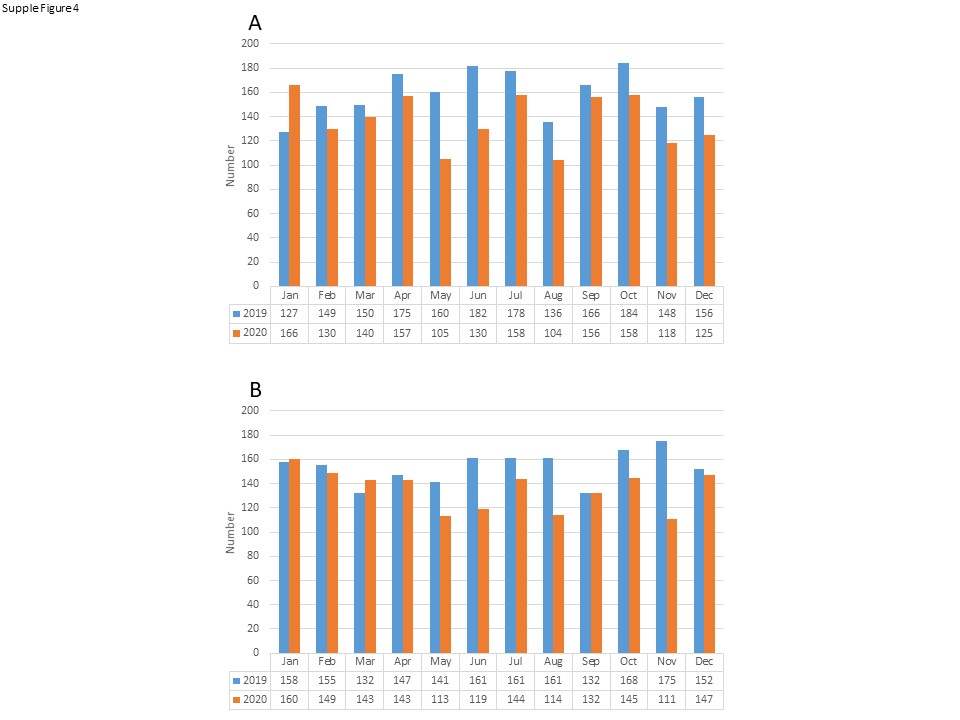

Supplement: Supplementary file 4 — Figure S4 Monthly number of diagnosed esophagogastric cancers (a) and colorectal cancers (b) in 13 institutes. [file DEO2-4-e249-s001.JPG]
